# Risk of SARS-CoV-2 Breakthrough Infection in Vaccinated Cancer Patients: A Retrospective Cohort Study

**DOI:** 10.1186/s13045-022-01290-8

**Published:** 2022-05-21

**Authors:** Anthony Rooney, Cory Bivona, Ben Liu, David Streeter, Han Gong, Qamar Khan

**Affiliations:** 1 University of Kansas Medical Center, 3901 Rainbow Boulevard, Kansas City, KS 66160 USA; 2 University of Kansas Health System, 4000 Cambridge Street, Kansas City, KS 66103 USA; 3 University of Kansas, 1450 Jayhawk Boulevard, Lawrence, KS 66045 USA

**Keywords:** SARS-CoV-2, COVID, Hematologic malignancy, Oncology

## Abstract

Although messenger RNA (mRNA) vaccines have established efficacy for prevention of severe SARS-CoV2 infection in the general population, their effectiveness in patients with malignancy, especially those on anti-neoplastic therapies, remains an area of open research. In order to better understand the risk of developing breakthrough SARS-CoV-2 infection and the outcomes associated with breakthrough infection for cancer patients, individual patient data from a curated outcomes database at the University of Kansas were retrospectively reviewed to determine the rate of breakthrough infection during an 8-month period encompassing the height of the delta variant surge. Although the rate of breakthrough infection in cancer patients after two doses of an mRNA vaccine remained low at 1.1%, hospitalization and death rates were 27 and 5%, respectively. Patients with hematologic malignancies, especially multiple myeloma, and those on anti-neoplastic therapy at the time of vaccination were found to be at higher risk for developing breakthrough infection.

To the editor,

Both the BNT162b2 (BioNTech, Pfizer) and mRNA-1273 (Moderna) vaccines have established efficacy for preventing severe SARS-CoV-2 infection [1, 2]. However, concerns remain regarding efficacy in patients with malignancy [3–5], especially those receiving anti-neoplastic therapies. To better elucidate the efficacy of vaccination in this patient population, we evaluated the frequency of breakthrough infection in vaccinated patients who received care at the University of Kansas Cancer Center.

The University of Kansas Cancer Center Curated Cancer Clinical Outcomes Database (C3OD) was queried for patients who had received either BNT162b2 or mRNA-1273 vaccines between 2/19/2021 and 10/31/2021. During this period, all patients receiving anti-neoplastic therapy were required to have SARS-CoV-2 testing via nasopharyngeal RT-PCR prior to each new treatment cycle. Patients not receiving anti-neoplastic therapy were screened for COVID symptoms and underwent nasopharyngeal RT-PCR testing if they screened positive. Patients who received both doses of either vaccine and had a subsequent positive SARS-CoV-2 nasopharyngeal RT-PCR swab during this time period were identified. Breakthrough infection was defined as a positive nasopharyngeal RT-PCR swab for SARS-CoV-2 > 14 days following completion of two doses of either BNT162b2 or mRNA-1273 vaccines. All patients with breakthrough cases had a confirmed negative SARS-CoV-2 nasopharyngeal PCR prior to breakthrough infection. Relative risk (RR) with 95% confidence intervals (95% CIs) was used to measure association of variables with breakthrough infection.

A total of 9417 patients received two doses of an mRNA vaccine and were included in this analysis. 68% received BNT162b2 and 32% received mRNA-1273. Median age was 67 years (range 13–100) and 57% of patients were female. 81% of the patients had a solid tumor and 19% had a hematologic malignancy. A total of 1490 (15.8%) patients received antineoplastic therapy during the observation period and 834 (8.9%) of patients received antineoplastic therapy at the time of vaccination. Median follow-up after second dose of vaccine was 224 days (range 0–319). One hundred and five patients had a breakthrough infection for a breakthrough infection rate of 1.1% (Table 1) and median time to breakthrough infection of 144 days (range 15–271) from completion of vaccination. 16% of patients with breakthrough infection remained asymptomatic, 51% had symptoms but were not hospitalized, 27% were hospitalized, and 5% died as a result of the infection. Breakthrough infections were more common in patients with a hematologic malignancy compared to a solid tumor (RR, 1.63; 95% CI, 1.0–2.57) and among patients who were receiving anti-neoplastic therapy (RR, 2.73; 95% CI, 1.71–4.34) (Table 2). Patients with multiple myeloma had the highest breakthrough infection rate (RR, 2.96; 95% CI 1.64–5.36), whereas patients with breast cancer had the lowest rate compared to all other groups combined (RR, 0.51; 95% CI 0.28–0.92).

**Table 1:** Frequency of breakthrough infection during observation period

	*n*	Frequency	95% CI (%)
All patients	9417	105 (1.1%)	0.9–1.4
BNT162b2 (2 doses)	6423	78 (1.2%)	1.0–1.5
mRNA-1273 (2 doses)	2993	27 (0.9%)	0.6–1.3
Solid tumor malignancy	6948	68 (1.0%)	0.8–1.2
Hematologic malignancy	1567	25 (1.6%)	1.0–2.4

**Table 2:** Association of variables with breakthrough infection

Variable		Relative risk (RR)	95% CI of RR
Age (years)	≤ 50	1.48	0.90–2.45
	51–65	0.86	0.56–1.31
	> 65	0.93	0.64–1.37
Sex	Male vs female	1.44	0.98–2.10
Vaccine type	BNT162b2	1.35	0.87–2.08
	mRNA-1273	0.74	0.48–1.15
Cancer type	Hematologic vs solid	**1.63**	**1.03–2.57**
On antineoplastic therapy at the time of vaccination	Yes vs No	**2.73**	**1.71–4.34**
Cancer subgroup	Breast	**0.51**	**0.28–0.92**
	Endocrine	0.42	0.10–1.69
	Gastrointestinal	1.17	0.63–2.18
	Genitourinary—female reproductive	1.91	0.94–3.91
	Genitourinary—male reproductive	1.01	0.49–2.07
	Genitourinary—unspecified male/female	1.31	0.61–2.81
	Head and neck	0.26	0.04–1.86
	Leukemia	1.06	0.47–2.41
	Lymphoma	0.94	0.44–2.06
	Multiple myeloma	**2.96**	**1.64–5.36**
	Respiratory	0.89	0.36–2.17
	Skin	1.21	0.69–2.12
	Unspecified	2.25	0.32–15.76

Bolded rows represent variables for which the 95% CI for RR does not cross 1.0

In summary, although the breakthrough infection rate in cancer patients during the observation period after two doses of an mRNA vaccine was low at 1.1%, infections were more severe with hospitalization and death rates of 27 and 5%, respectively, highlighting the need for ongoing infection prevention and vigilance among this population. Patients with hematologic malignancies have lower rates of seroconversion following vaccination [6], and there has been concern that this patient population remains at increased risk for breakthrough infection. The higher rates of breakthrough infection in patients with hematologic malignancies noted in our analysis validate these concerns. In particular, patients with multiple myeloma were at higher risk for breakthrough infection. Whether this increased risk is due to underlying disease biology or pharmacologic immunosuppression remains an open question. Patients with hematologic malignancies, especially those with multiple myeloma, and those receiving anti-neoplastic therapies should be counseled on an ongoing basis about their risk of infection and prioritized for receipt of newly developed pre- and post-exposure therapeutics directed against SARS-CoV-2.

## Data Availability

The datasets used and/or analyzed during the current study are available from the corresponding author on reasonable request.

## Abbreviations

C30D: Curated Cancer Clinical Outcomes Database
RT-PCR: Real-time polymerase chain reaction
RR: Relative risk
CI: Confidence interval
mRNA: Messenger ribonucleic acid
